# Milk bioactives may manipulate microbes to mediate parent–offspring conflict

**DOI:** 10.1093/emph/eov007

**Published:** 2015-04-02

**Authors:** Cary R. Allen-Blevins, David A. Sela, Katie Hinde

**Affiliations:** ^1^Department of Human Evolutionary Biology, Harvard University, Cambridge, MA 02138, USA; ^2^Department of Food Science, University of Massachusetts, Amherst, MA 01003, USA; ^3^Department of Organismal and Evolutionary Biology, University of Massachusetts, Morrill Science Center, Amherst, MA 01003, USA; ^4^Brain, Mind, and Behavior Unit, California National Primate Research Center, UC Davis, Davis, CA 95616, USA

**Keywords:** human milk oligosaccharides, commensal bacteria, microbiota, maternal investment, infant development, lactation

## Abstract

Among mammals, milk constituents directly influence the ecology of the infant’s commensal microbiota. The immunological and nutritional impacts of breast milk and microbiota are increasingly well understood; less clear are the consequences for infant behavior. Here, we propose that interactions among bioactives in mother’s milk and microbes in the infant gut contribute to infant behavioral phenotype and, in part, have the potential to mediate parent–offspring conflict. We hypothesize that infant behavior likely varies as a function of their mother’s milk composition interacting with the infant’s neurobiology directly and indirectly through the commensal gut bacteria. In this article, we will explore our hypothesis of a milk-microbiota-brain-behavior dynamic in the context of the coevolution between human milk oligosaccharides, bacteria, the gut–brain axis and behavior. Integrating established features of these systems allows us to generate novel hypotheses to motivate future research and consider potential implications of current and emerging clinical treatments.

## INTRODUCTION

**Figure 1.**
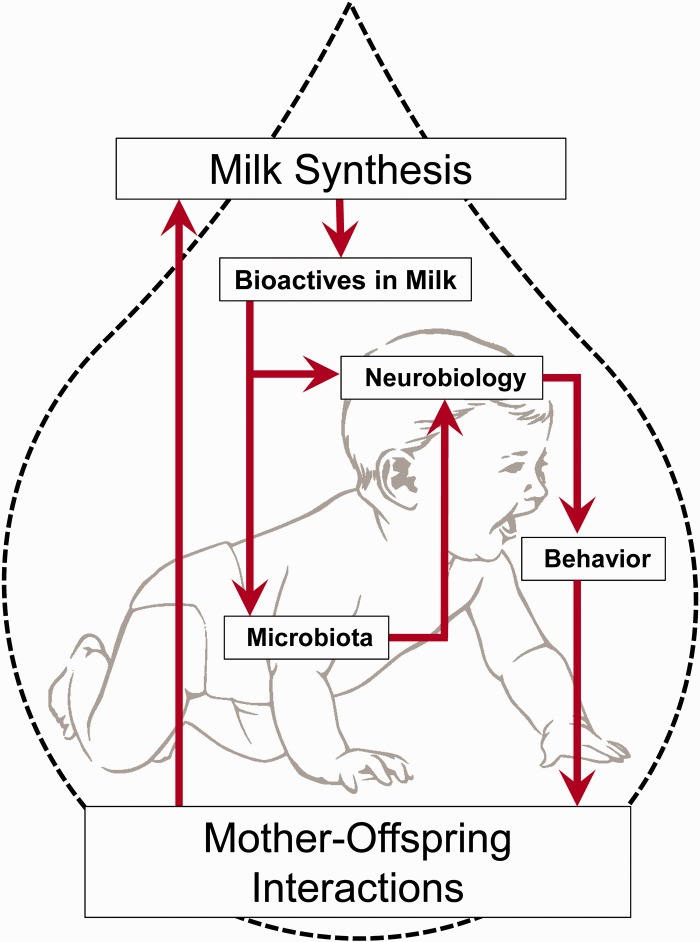
Conceptual model of bioactives in milk mediating maternal-offspring conflict and coordination. Bioactives in milk affect gut microbiota in the infant, impacting the development of neurobiology and subsequently behavior.

Mammalian infants are reliant on their mother’s milk for survival, as are their coevolved gut microbiota. Infants are primarily exposed to complex microbial communities perinatally during vaginal birth and throughout infancy, with milk constituents directly influencing the ecology of the infant’s commensal microbiota [1–4]. Within this exquisitely complex, dynamic system, hundreds of bacterial species and a wide array of milk components interact, affecting immune function and bioavailability of nutrients. Research effort has predominantly, and understandably, been directed to the immunological and nutritional impacts of breast milk and microbiota from both clinical and evolutionary perspectives [4–9]. Meanwhile, the intersections among mother’s milk, microbial ecology and the gut–brain axis, and the consequences for infant behavior, have yet to be investigated. Here, we propose that interactions between bioactives in mother’s milk and microbes in the infant gut contribute to infant behavioral phenotype and, in part, have the potential to mediate parent–offspring conflict and coordination (Fig. 1). We hypothesize that infant affect and behavior—crying, suckling, activity, emotionality—in breastfed infants likely varies as a function of their mother’s milk composition interacting with the infant’s neurobiology and physiology directly and indirectly through the commensal gut bacteria. In this article, we will explore our hypothesis in the context of the coevolution between human milk oligosaccharides (HMO) and bacteria, with implications for neonatal brain and behavior. In addition, we will consider potential interactions with maternal-origin hormones, previously demonstrated to affect offspring biobehavioral organization. Integrating established features of these systems allows the generation of novel hypotheses to motivate future research, especially in light of potential clinical implications and applications.

## THE COMPLEXITY OF MOTHER’S MILK

Mother’s milk is a complexly structured, highly personalized biological fluid transferring bioactive constituents to the developing neonate [1, 10, 11]. The presence and relative abundance of individual milk bioactives vary as a function of maternal genetic, pathogenic, somatic, life-historic, phylogenetic and environmental characteristics. In addition to the calories from macroconstituents, mother’s milk also provides the infant with immunoglobulins, minerals, hormones and oligosaccharides [11]. Milk oligosaccharides are variably structured chains of sugars with a lactose core [10, 12]. These complex sugars are found across mammalian taxa, but the oligosaccharide profiles in milk are highly variable among species [12]. In humans, milk oligosaccharides are the third most plentiful component of milk and likely represent a substantial proportion of lactation effort.

The diversity, complexity and abundance of HMO show evidence of divergence from other primates, suggesting the oligosaccharides in human milk have been a target of natural selection [11, 13]. The particular profile of oligosaccharides an individual mother produces is variable, heritable and can be associated with fitness proxies [7, 12, 14, 15]. To date, hundreds of oligosaccharides have been identified in human milk [16]. Humans produce a greater diversity and higher abundance of oligosaccharides than do any of the apes, monkeys or strepsirrhines investigated to date, typically by an order of magnitude [11]. Despite the diversity of potential HMO, individual mothers produce only a subset, generally ∼50 [17]. This subset, or HMO profile, varies by presence, abundance and proportion of particular HMO isomers. One known predictor of the HMO profile a mother produces is her secretor status, determined by specific alleles encoding fucosyltransferases (e.g. *FUT2* gene) that attach fucose sugars to HMO, creating fucosylated HMO [10, 17]. Mothers without secreter alleles, known as non-secretors, produce a much more limited amount of fucosylated HMO [15, 18]. Additionally, HMO profiles change over the course of lactation—the presence and prevalence of particular isomers shift while the total proportion of HMO in milk declines [15, 18].

## MOTHER’S MILK AND INFANT GUT MICROBIOTA

Mothers’ HMO profiles have been associated with the establishment and maintenance of commensal bacteria [3, 4, 19, 20]. HMO are not primarily digested by the infant for nutrition as they remain largely intact during passage to the colon [4]. Once in the colon, HMO can be metabolized by intestinal microbiota able to enzymatically cleave the HMO bonds [3, 4]. As the infant’s gut matures, ecological succession occurs and anaerobic bacteria belonging to genera such as *Bifidobacterium* and *Bacteroides* become numerically dominant [3]. Notably, select bifidobacterial genomes contain unique gene clusters that enable efficient HMO metabolism [21, 22]. This is consistent with their frequent overrepresentation in the infant gut microbial community [22]. In addition, *Bacteroides *possess mucus utilization pathways to consume structurally similar soluble HMO [23]. The ability of the infant’s commensal gut microbiota—but not the infant’s endogenous enzymes—to digest milk oligosaccharides suggests that mothers are feeding bacteria, too.

HMO profiles also influence infant susceptibility to viral and bacterial pathogens [3, 18, 24]. Milk oligosaccharides passing through the infant’s gastrointestinal tract can bind to virus or bacterial strains like rotavirus, *Escherichia coli*, *Campylobacter jejuni* and *Vibrio cholerae* [4, 18, 20]. Protection from these diarrheal diseases, a leading cause of infant mortality, is expected to be a major selection pressure [25]. Fucosylated HMO are better ‘decoys’ for *Camplyobacter*, *E.coli* and norovirus, as the fucosyl portion of the molecule are similar to those presented on the gut epithelium to which pathogens bind [24, 25]. However, non-fucosylated HMO also protect against serious pathogens, like rotavirus [25]. Additionally, HMO discourage the establishment of pathogens in the gut through supporting the growth of specific bacteria. The beneficial bacteria attached to the gut epithelium become competitive inhibitors of pathogenic invaders, protecting the infant from disease [22].

## MICROBIAL FUNCTIONS IN THE INFANT GUT

The community structure of the infant gut, shaped in part by mother’s milk, is instrumental for the infant’s physiological development [3, 20, 26]. The gut bacteria are important for programming early immune responses, bioconverting ingested nutrients and inhibiting pathogenic bacteria [20]. Gut microbiota synthesize vitamins necessary to the host and ferment carbohydrates that are otherwise indigestible [22]. Metabolism of such carbohydrates may also increase the bioavailability of minerals, like iron, to the host [27]. Bacteria in the gut also produce short-chain fatty acids capable of crossing the blood–brain barrier and impacting the synthesis of neurotransmitters [28]. Exposure to gut microbiota in infancy also appears to ‘prime’ the immune system and disturbances in early gut microbiota have been associated with auto-immune and allergic diseases [8]. The microbial transfer from mother to offspring is an important aspect of natal development that continues into infancy as microbes are fed by the mother [29]. The infant intestinal microbiome and the potentially adaptive capacity to synthesize HMO likely coevolved in response to selective regimes that exerted particularly strong pressures on immunity, nutrient intake and the mother–offspring relationship during infancy in human evolution.

## BODY AND BRAIN

### Gut–brain axis

**Figure 2.**
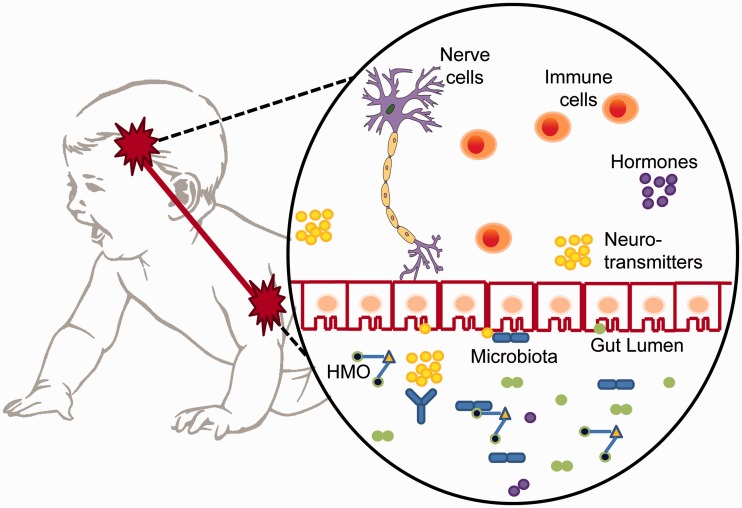
The gut–brain axis pathways by which gut microbiota can affect neurobiology and subsequently behavior. Bacteria (blue rods and olive green circles) can produce neurotransmitters (yellow circles) or extract them from the gut lumen. Neurotransmitters can then interact with nerve cells of the vagus nerve or be released into portal circulation and possibly interact with other nerve cells. Microbiota can induce immune cell (red circles) activation or release hormones (purple circles). Bacterial species can also competitively inhibit other species, effectively selecting the metabolites able to be produced in the gut.

The gut and brain communicate in bidirectional pathways along the gut–brain axis (Fig. 2). Although the main signaling route between the gut and brain is the vagus nerve connecting the enteric nervous system to the brain, immunological and hormonal interactions also exist [9]. Aberrant assembly of intestinal microbiota (i.e. dysbiosis) can activate an inflammatory response that induces depressive-like sickness behaviors and impairs cognition [30]. Gut microbiota can also release molecules that function as neurotransmitters in their host, like catecholamines [9, 31]. Catecholamines produced by the host also affect the gut, as indicated by a seven log-fold rise in *E.coli* after systemic release of catecholamines in response to neurotoxin administration [31]. Exposure to microbes also increases the cannabinoid and opioid receptors in the rodent intestine [9]. These neurological and endocrine pathways of the gut–brain axis develop in the first 1000 days of life, as brain size doubles, cortical neurogenesis occurs, the hypothalamic–pituitary–adrenal (HPA) axis becomes regulated and the mucosal barrier of the gut strengthens [2, 6, 7, 32, 33].

### HPA axis

The HPA axis controls the release of the glucocorticoids that are instrumental in metabolic, immune and biobehavioral responsivity and regulation [34, 35]. The HPA axis is an endocrine cascade: corticotrophin-releasing hormone is secreted from the hypothalamus, which binds to receptors in the anterior pituitary stimulating the release of adrenocorticotropin hormone (ACTH) that then passes into circulation and stimulates the secretion of glucocorticoids from the adrenal glands [26, 32, 34]. Acute upregulation of the HPA axis occurs when an organism is confronted by situational challenges and motivates the ‘fight or flight’ response, as well as orchestrates accompanying metabolic, immunological and behavioral responsivity. At baseline, however, glucocorticoids follow a diurnal rhythm and maintain essential homeostatic functions in the body, such as catabolism of fat stores [32, 35]. Importantly, the HPA axis underlies stable individual differences in behavioral phenotype, known variably as personality, temperament and behavioral syndromes [36]. In this way, the HPA axis importantly underlies the biobehavioral regulation of individuals. Moreover, signaling pathways originate in the brain and glucocorticoid receptors are found in tissues throughout the body, including the intestinal tract, creating multiple intersections between the HPA and gut–brain axis. The concentration and expression of glucocorticoid receptors in the intestinal tract are particularly high during infancy when infants are receiving maternal-origin glucorticoids via milk and are seemingly co-organizing neurobiological and behavioral development [35–37].

## INTERSECTING PATHWAYS OF THE HPA AND GUT–BRAIN AXES

Interactions between the HPA and gut–brain axes have been demonstrated in adult germ-free rodent models that display aberrant behavioral phenotypes [38]. Germ-free mice have higher plasma levels of ACTH and glucocorticoids in response to being physically restrained—a stressful manipulation—compared with specific-pathogen free (SPF) mice [39]. However, gnotobiotic mice that were colonized only by *Bifidobacterium longum *subsp*. infantis* exhibited glucocorticoid secretion equivalent to the response of SPF mice [39]. The increased glucocorticoid response in germ-free mice is also alleviated by inoculation with SPF feces; however, this intervention was only effective at juvenility and possibly transitions to adulthood [39]. From these studies, we can infer neurological development of the animal must occur in concert with colonization of the microbiota to have effects on certain aspects of biobehavioral phenotype [2, 40]. Among humans, the first 1000 days is a sensitive period of intense maternal effort and critical developmental windows during which infants are particularly sensitive to environmental and maternal conditions [41]. During infancy, maternal-origin hormones ingested via milk shape growth, development and behavior [36, 42]. Concurrently, maternally and environmentally transferred microbes colonize the infant, partly as a function of milk oligosaccharides [4]. As such, the HPA axis, the gut–brain axis, their intersections and their influences on infant behavior are likely shaped by mother’s milk.

## MICROBIAL INFLUENCES ON HOST BEHAVIOR

The community structure of gut microbiota influences host behavior [9]. Research with gnotobiotic mice has demonstrated that gut microbial colonization affects social and anxiety-like behavior [33, 40, 43]. Germ-free mice without intestinal microbiota deviate from species-typical behavior; they are less social and do not prefer novel over familiar mice [40]. Additionally, species-typical microbiota colonization manifests a more exploratory, less nervous behavioral phenotype than displayed by germ-free mice [44]. Colonizing adult rodents with one or two strains of *Bifidobacterium* spp. or *Bacteroides* spp. can improve behavioral phenotype, including a partial recovery of social behavior, reduced anxiety and decreased stereotyped behavior [43, 45, 46]. This is particularly salient given that several species belonging to these two genera have been demonstrated to metabolize HMO [23].

Conversely, for the individual, challenging experiences can alter gut microbial ecology with persistent effects months later [28]. *Bifidobacteria* and *Lactobacillus* populations are reduced in infant rhesus macaques stressed prenatally by maternal exposure to an acoustic startle [47], suggesting these bacterial genera may be especially vulnerable to host stress. Given bifidobacteria’s sensitivity to host stress, it may be advantageous that its host remains calm, potentially explaining the role of some strains in reducing anxious behavior and stress reactivity in the host [48]. Maternal separation models have also induced changes in offspring microbial ecology, including the reduction of *Lactobacillus* [28].

Milk bioactives encourage the growth of specific gut bacteria that may produce particular behavioral phenotypes. Infants with colic, a syndrome marked by extensive crying, have less diverse microbiota than healthy infants [49]. However, symptoms of colic were significantly reduced after inoculation with lactic acid bacteria, specifically *Lactobacillus reuteri* [22, 50]. *L.reuteri* also reduces the duration of acute infectious diarrhea in infants and children [51]. Infants with higher concentrations of bifidobacteria in their gut also exhibited less crying and fussing in the first 3 months of life [52]. Although these effects may be due in part to alleviation of gastrointestinal distress, we speculate that HMO in mother’s milk may function to promote the colonization of microbiota that influence offspring biobehavioral regulation in concert with immune and nutritional effects.

There has not, to our knowledge, been a published study that experimentally or observationally investigated the potential effects of HMO or other milk bioactives on the microbial community structure/function and resultant behavioral phenotype in model organisms or humans. Recently, microbiota composition and temperament have been associated in children aged 18–27 months [53]. Breastfeeding duration, as a dichotomous variable, did not have a significant effect on temperament factors associated with gut microbial profiles; however, the presence and abundance of HMO or other ‘biobehavioral’ milk bioactives were not examined [53]. Nonetheless, this research represents a crucial step forward, as the majority of studies demonstrating microbial effects on behavior have been performed on weaned animals, despite evidence that colonization within critical early windows is necessary to affect behavioral development [39, 40].

## AN EVOLUTIONARY PUSH–PULL: PARENT–OFFSPRING CONFLICT

Mammalian mothers and infants engage in complex behavioral and physiological negotiations to determine the amount and duration of maternal care and milk transfer. Parent–offspring conflict is the expectation of an essential tension between mothers and infants in the preferred amount of maternal investment that is predicated on their divergent genetic interests [54–57]. All else being equal, and acknowledging that rarely is that the case, natural selection is expected to have shaped adaptations operating in mothers to equally allocate resources toward multiple offspring across a reproductive career to maximize lifetime reproductive success [56]. In contrast, the infant being entirely related to himself, but sharing fewer genes with the mother and siblings, is expected to manifest adaptations to extract more resources from the mother than she is adapted to provide or to use resources according to his self-interest [56]. This conflict can be evident in short-term mother–infant interactions of infant signals for investment, in how the infant utilizes maternal investment and the duration of time until infant independence [36, 54, 56, 58]. For example, infant behavioral tactics for eliciting maternal care and milk can be both positive and negative stimuli—such as nuzzling, smiling, crying and tantrums [54, 59].

As infants age, parent–offspring conflict is expected to intensify as, with each increment of investment that is provided, the benefit to the mother is reduced in terms of infant survival and improved condition [56]. Although the direct fitness of either increases the inclusive fitness of the other, such that they each benefit from coordinating behavioral care and physiological investment, the divergence of infant demand optima and maternal supply optima increases as infants age [36, 41, 54]. Infants are increasingly more costly; they are bigger and more active so their energetic requirements are greater. To meet that demand, the physiological costs of milk synthesis increase, diminishing maternal reserves and potentially extending the period of recovery until mothers can support subsequent reproduction [35, 60]. As infants age, they can begin to exploit solid foods and are not entirely dependent on maternal nutritional support. As such, from the mother’s perspective, costs are increasing and benefits are decreasing [56, 58]. Mothers, depending on their physical condition or stage of their reproductive career, may have more incentive to have lower daily costs of infant rearing or truncated duration of investment [60]. For example, adolescent mothers with fewer resources to sustain lactation may benefit to a greater extent if their infants have relatively lower daily energetic costs or behavioral demand [36].

Mothers are expected to have coevolved countermeasures to infant demands for and utilization of investment, possibly through milk [36, 41, 54, 58]. Milk bioactives have been implicated in shaping infant behavioral phenotype, possibly to more optimally allocate maternal energetic investment. Collectively, this area of research reveals critical windows of biobehavioral organization, in part sexually differentiated, and influenced by mother’s milk. Experimentally elevated glucocorticoids ingested via mother’s milk demonstrate behavioral and neurobiological effects persisting into adulthood [61]. Among rhesus monkeys, glucocorticoids in milk may contribute to orchestrating infant trade-offs between growth and behavioral phenotype [36]. Younger, smaller and less-experienced mothers produced lower available milk energy but higher cortisol concentrations in milk. The cortisol signal in milk, independent of available milk energy, predicted a behavioral phenotype characterized as more nervous and less exploratory but had greater daily weight gain during infancy [36]. Speculatively, hormonal signals in milk may shape infant developmental priorities, influencing infant physiology to allocate milk energy to growth rather than expensive behavioral activities like play and exploration [36].

## THE DOUBLE-EDGED SWORD OF MILK OLIGOSACCHARIDES?

The established HMO-microbial interactions, reducing pathogenic infection and improving nutrient availability in the infant, arguably function to enhance return on maternal investment. We hypothesize along similar lines that HMO in part shape infant microbial communities to shift infant behavioral phenotype toward maternal investment optima by reducing the costs of rearing the infant. If milk is mediating maternal-offspring conflict through behavioral effects of the nascent gut microbiota, we can make several testable predictions (Table 1). Particular HMO isomers or HMO profiles may program the establishment of microbes that exert biobehavioral effects. We would expect that HMO would particularly target multifunctional bacterial strains that contribute to immunocompetence and nutrient bioavailability for the infant as well as behavioral manipulation toward the mother’s optima. In such a situation, there would be no added cost to the mother to produce the HMO to manipulate the infant’s behavioral phenotype, and the yoked benefits to the manipulation would constrain the evolution of infant countermeasures [62].

**Table 1.** Hypotheses and predictions for infant behavioral phenotype from maternal, infant and microbial interactions. Integrating parent-offspring conflict theory, across life history and ecological contexts, we predict variable manifestations of infant behavioral phenotype as mediated through microbial influences on the brain.

Perspective	Hypotheses and Predictions	
Maternal	**(1) Mothers are expected to favor a less costly infant phenotype.**	
	An infant behavioral phenotype that is less energetically costly in terms of maternal caloric transfer could manifest as:	
	A	Less demanding: elicits less maternal behavioral care, e.g. decreased suckling, crying
	B	Less energy expenditure: a temperament that has a lower daily energetic budget, e.g. less locomotion, play, exploration
	C	Earlier age of independence: less time to weaning threshold, increased reliance on allomothers, faster attainment of social and foraging skills, more ‘confident’ temperament
	Across social, nutritional and ecological contexts:	
	Reduced energy expenditure is predicted to be particularly favored in risky environments characterized by infectious disease, injury and predation	
	Reduced energy expenditure and less demanding behavioral phenotypes expected to be favored under conditions of low food availability due to ecology (population) and/or access to resources (individual)	
	Maternal optima in cooperative breeding or biocultural reproduction systems are expected to favor earlier age of independence from maternal resources and/or more demanding behavior directed to non-mothers.	
	Across life history:	
	Young/early reproductive career mothers that are still growing are expected to particularly favor lower infant energy expenditure.	
	Prime condition and mid-career mothers are expected to favor an infant behavioral phenotype of earlier independence to shorten inter-birth intervals.	
	Mothers favoring an infant phenotype of earlier independence will have an increased metabolic cost at peak lactation but a faster return to cycling, compared with mothers programming for reduced energetic cost	
	**(2) Milk composition influences microbial communities that shape infant behavioral phenotype.**	
	Mothers predicted to favor particular infant behavioral phenotypes (A, B or C) will produce, in part, differentiated milk oligosaccharide profiles.	
	Particular milk oligosaccharide profiles are expected to differentially promote the colonization and maintenance of microbial communities that affect gut–brain axis regulation and infant neurobiology.	
	Infant gut microbiota shaped by milk oligosaccharides are predicted to influence regions of the brain underlying emotion regulation and behavioral motivation to influence a less costly behavioral phenotype	
Infant	**(3) Infants are expected to exhibit some counter-tactics to milk-microbiome-mediated influences on behavior**	
	As infants mature, infant gut physiology becomes less hospitable to milk-oriented microbiota that exert behavioral influences toward maternal optima.	
	Infants will increase their exposure to non-maternal bacteria through environmental exposure and supplemental food to reduce the behavioral influence of milk-oriented microbiota.	
	Insofar as HMO profiles influence bacteria that simultaneously improve immune response, nutritional bioavailability and behavioral phenotype, infants may be limited in countering maternal influences on behavioral phenotype, particularly during early infancy.	
Microbial	**(4) Bacterial influence on infant behavioral phenotype is dependent on bacterial species and phase of infancy.**	
	In early infancy, milk-oriented microbiota in the infant gut will produce a less energetically costly behavioral phenotype.	
	As weaning progresses and milk-oriented microbiota receive less milk, these bacteria will neurobiologically motivate milk demanding behaviors, such as tantrums.	
	Microbiota that can consume milk oligosaccharides, host glycans and molecules from complementary food will influence an earlier independence behavioral phenotype to pursue non-maternal foods.	

Relevant citations: theoretical motivation: 2, 3, 15, 18, 21, 23, 30, 35, 36, 44, 52, 60, 63, 64–67; relevant empirical research: 2, 9, 19, 20, 35, 36, 56, 58, 59, 68, 69, 70–72

Complicating the dynamics further, particular HMO isomers or classes of HMO may not necessarily be the target of selection for influencing behavioral phenotype alone; rather, combinations of HMO acting in concert may be critical to shift microbial ecology toward maternal optima. Such shifts may include increased *Bifidobacteria*, *Bacteroides* spp. or *Lactobacillus* spp. previously implicated in behavioral outcomes [22, 43, 45, 46]. Alternately, behavioral changes may not come from a strict increase in a particular bacterial group; but rather, proportionate changes may have greater impact. For example, two infants may have the same amount of bifidobacteria in their gut, but the overall proportion of bifidobacteria relative to their other gut microbiota could be very different. The proportionate interactions with other bacterial genera or species could drive differences in behavioral phenotype.

## PARENT–OFFSPRING CONFLICT ON A MICROBIAL LANDSCAPE

**Figure 3.**
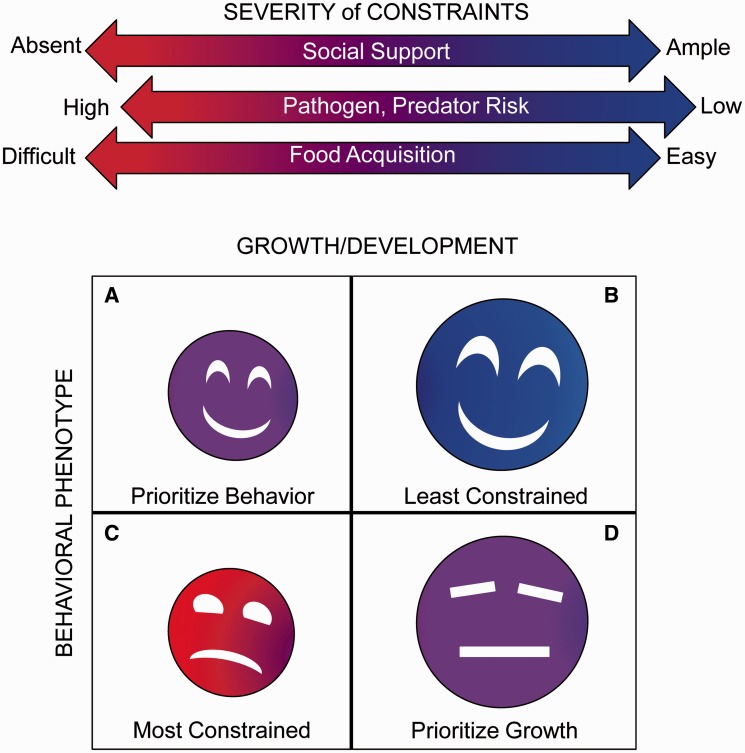
The relationship between constraints and less costly phenotypes. The intensity of constraints (red indicates severe; blue indicates relaxed) on the mother–infant dyad affects the definition of a ‘less costly’ phenotype from a maternal resource perspective. Under situations of mild constraints (**A** and **D**), less costly phenotypes will prioritize growth or behavior because resources are not available to prioritize both. Under severe constraints (**C**), less costly phenotypes will be delayed in both growth and behavior. Under relaxed constraints (**B**), resources can be allocated to behavior and growth.

Commensal infant bacteria, operating closer to maternal optima, may influence infants to acquire or require lower daily investment or shorter duration of investment (Table 1). Mothers would be expected to provide less behavioral care and transfer less milk energy to infants who exhibited fewer signals to elicit maternal investment (less distress, crying and suckling intensity) [58]. Additionally, reduced energetic expenditure (less locomotion, exploration and play) could either decrease daily caloric demand or allow infants to prioritize growth, thus reaching weaning thresholds earlier [73]. Among cooperatively breeding species, like humans [74], a less costly behavioral phenotype may increase demand for investment from non-maternal caretakers, while decreasing demand for maternal investment. For example, a human infant may exhibit increased smiling, laughter or cuddling but decreased suckling. Cooperatively breeding species may also engage in allo-maternal nursing, where females provide milk for infants not their own. Although such a practice might be expected to disrupt the hypothesized system, the volumes of allo-maternal milk necessary to swamp the maternal effect would need to be substantial [75]. Maternally influenced biobehavioral microbes may also accelerate behavioral development and earlier independence from the mother but would be less likely in risky nutritional, disease, social or predator ecologies (Fig. 3). Mothers limited in their capacity to synthesize milk or sustain lactation—young, nutritionally marginal, unhealthy or otherwise constrained—may particularly benefit from infants characterized by a less costly behavioral phenotype. A less costly infant phenotype that is shifted toward the maternal optima is expected to have measurable effects, including accelerated somatic recovery during the weaning process, faster returns to cycling, shorter inter-birth intervals and higher probabilities of successful subsequent pregnancies.

Infants may be limited in their counteradaptations to maternally influenced microbial manipulation, especially at younger ages. Currently, to the best of our knowledge, no pathways have been described through which infants have the capacity to influence the HMO profile of the mother. Moreover, if milk oligosaccharides are instrumental for infant health and nutrition, constraints while the immune system is naïve and alternative nutrition is unavailable may prohibit selection favoring infant countertactics. However, the infant’s gut physiology is partially self-organizing, not solely dependent on maternal and bacterial input [76], opening the door for countermeasures to discourage the growth or dominance of microbiota that provides a maternal advantage, especially as infants mature. Exposure to non-maternal microbiota could also lessen the impact of maternal manipulation. Infants are exposed to other microbes through environmental exposure and complementary foods, especially as they mature [4, 77].

There is a third party to this parent–offspring conflict model: the microbes. The gut microbiota has its own self-preservation interests that must be considered [9]. A microbe that increases maternal fitness is more likely to be carried by the mother and be vertically transmitted to the infant [29]. These microbes can then benefit the infant through building immunity and providing nutrient bioavailability, increasing the fitness of the infant. However, the microbe’s influences on the infant will also be a target of selection insofar as they influence the microbes’ fitness. Although it is counterintuitive to predict milk-oriented microbes would influence behavior to reduce the demand for milk, microbes that are milk-oriented, such as *Bifidobacteria infantis*, may have evolved a trade-off: these microbes program for a less energetically costly phenotype on a daily basis with the potential to prolong their milk exposure across infancy. During the weaning process, the gut undergoes dramatic shifts in microbial composition because of less milk consumption [2]. As their numbers decline, milk-metabolizing bacteria may release toxins or neurochemicals in the absence of HMO [9]. Toxins interacting with the gut–brain axis may cause an increase in care-eliciting behaviors, in an effort to increase milk delivery to the infant and its microbiota; and these may partially explain the magnitude of weaning tantrums [9, 56]. Additionally, microbes that can metabolize HMO and host mucus glycans or other carbohydrates, like *Bacteroides* spp. [9, 23], could program for an earlier independence phenotype. Because the fitness of these microbiota is less dependent on the presence of HMO, they may not contribute to an increase in care-eliciting behaviors at weaning.

## EXPERIMENTAL APPROACHES TO INVESTIGATE MECHANISTIC PATHWAYS

A multifaceted research approach, including *in vitro* cultures, animal models and human studies, will be necessary to systematically investigate a milk-microbiota-brain-behavior (M2B2) system (Box 1; Table 2). Identifying target ‘biobehavioral’ bacteria that are likely to be secreting neurotransmitters is a paramount first step [31]. Within this study system, bacteria isolated from the infant microbiome or ‘milk-oriented microbiota’ would provide the initial research target [24]. Infant-harbored microbial communities, including unculturable populations, could be examined via metagenomics to potentially determine their ability to secrete neurotransmitter-like molecules and their capacity to metabolize milk constituents. Bioactive milk components can also be identified by *in vitro *testing with bacterial strains and *in vivo* research in model organisms [81]. Experimental administration of milk-derived molecules to dams, to be secreted in milk, instead of directly treating neonates, is less invasive and may have fewer stress confounds, an important consideration for behavioral studies. Following identification of target milk constituents and bacteria, gnotobiotic animals can be inoculated with the target bacterium, treated with the bioactive and administered biobehavioral assessment [31]. Inoculation of dams or neonates with specific, singular microorganisms and bioactives will provide causal mechanistic pathways.

**Table 2.** Research priorities and pathways for an integrative understanding of the milk-microbe-brain-behavior (M2B2) system. Established and ongoing research currently addresses all elements of this system, but their integration provides new opportunities to understand adaptations in mothers and infants for negotiating conflict and coordination of maternal investment and infant utilization of that investment.

Topics	Agenda	Methods/disciplines	Measures
Milk oligosaccharides	Describe presence, abundance, sources of variation in milk oligosaccharides profiles intra-individually, inter-individually, across populations, across species	Analytical Chem, Biochemistry, Pediatrics, Animal Science	Milk sampling, longitudinal
Microbiota in milk	Identify mode of entry to milk, explore possibilities of selective translocation of maternal bacteria, determine whether milk microbiota survives passage through the stomach	Microbiology, Metagenomics, Physiology	Cultured, sterile biopsy of mammary tissue with analysis of maternal and infant gut microbiota
Infant microbiota	Determine presence, abundance of microbes longitudinally, response to perturbations	Microbiology, Metagenomics, Metabolomics	Fecal samples, 16S rRNA analysis, Cultures, Metabolic products
Milk-oriented microbiota	Identify microbiota capable of metabolizing human milk	Microbiology, Metagenomics, Biochemistry	*In vitro* cultures, metagenomic analysis of unculturable organisms
Infant behavior	Assess behavioral phenotype during the period of maternal nutritional dependence, the weaning process	Ethology, Behavioral Ecol, Anthropology Psychology, Human Biology	Activity level, affect, surgency/extraversion, vocalization, time spent on mother, time spent near conspecifics
Infant brain	Evaluate neural function, receptor density and structures in brain regions underlying emotion regulation and behavioral motivation.	Neurobiology, Biopsychology, Animal Science	*In vivo* neuroimaging, *ex vivo* receptor staining, neural mapping of regions of interest
Infant gut epithelium	Quantify receptor density and gene expression within the gastrointestinal tract to determine gut–brain axis pathways affecting emotion regulation and behavior.	Physiology, Animal Science	histology of neurotransmitter receptors, RNASeq of tissue in fecal samples
Maternal outcomes	Measure maternal recovery and transitions to subsequent reproduction in relation to infant behavioral phenotype	Evolutionary Anth, Human Biology, Behavioral Ecol	Metabolic cost of lactation, inter-birth interval, duration of amenorrhea, subsequent pregnancy outcome

Behavioral phenotype is necessarily mediated through the brain, so neural regions that underlie the development and maintenance of emotion regulation and behavioral motivation, generally midbrain areas, are likely to be implicated in the hypothesized behavioral effects. Specifically, the hypothalamus, anterior cingulate cortex, amygdala, insula and hippocampus, already known to be integral components of the gut–brain axis and developmentally sensitive, are important targets of future research of a M2B2 dynamic [44, 68, 82]. Similarly, the serotonergic system, implicated in anxiety and depression, is differentially regulated in germ-free mice [44, 69]. Moreover, early life experiences organize these brain systems and influence offspring behavioral phenotype [83, 84]. Maternally influenced biobehavioral microbes are likely to directly influence these regions through the infant’s gut–brain pathway, but also indirectly through shaping the behavioral experiences of their host.

**Box 1.Models of gut microbiota**

Much of our understanding of mammalian-microbe interactions emerges from biomedical research on animal models such as rodents and, to a lesser extent, pigs. Gnotobiotic animals are purposely colonized by a defined set of specific bacteria or remain germ-free having been raised and maintained in a sterile environment [4]. Models may be SPF, known to be free of particular microbial strains [78]. Of particular utility, gnotobiotic animals may be inoculated with human microbiota, producing ‘humanized’ models [79]. Using gnotobiotic animals permits controlled experiments to target the molecular mechanisms and functional outcomes of milk constituents interacting with resident microbiota. In contrast to gnotobiotic models, conventional models maintain a microbial ecology that is not experimentally composed prior to experimentation, but manipulated indirectly through exogenous interventions. This may include controlled diet, stress challenges and disease state that may prompt a characteristic shift in microbial ecology in form and/or function [43, 63, 80]. Animal models have been essential for understanding the mechanisms by which microbial products in the gut communicate with the brain via the gut–brain axis and provide important avenues for investigating mother’s milk, microbial ecology and infant behavior.

Proposed experiments are necessarily simplified from the naturalistic circumstances consisting of hundreds of milk bioactives and hundreds of microbes, but through systematic elaboration, researchers can address more complex interactions. Like the microbiota, milk bioactives may only have certain effects in concert with other milk components or behavioral care interactions. Observational and epidemiological studies that grapple with these complexities facilitate correlative patterns that can be evaluated for consistency with and departure from experimental findings. Although cross-population studies of human breast milk are often characterized by limitations in determining causality as well as methodological obstacles, logistical complexities and ethical considerations [85], they are necessary to situate milk bioactives and gut bacteria within their human evolutionary context. Employing collaborative, integrative, multifactorial approaches are especially important with microbiome studies, because the cooperation and antagonism between microbial taxa may be a driving force in colonization and microbial function [19].

## CONSIDERATIONS FOR HUMAN HEALTH

Current clinical practices can directly and indirectly influence the presence and abundance of commensal microbes during critical windows of developmental co-organization of multiple physiological systems in the infant [1, 4]. Cesarean deliveries, formula feeding and early administration of antibiotics, all of which can dramatically alter microbial community ecology and therefore the infant, are increasingly commonplace in the United States and around the world [86, 87]. Perturbations or dysbiosis of the early gut microbiota could have unexpected and persistent effects, including altered biobehavioral regulation, immunological function and metabolic processes [26, 29, 88, 89]. Ecological stressors, mediated through interaction with the mother, influence early development and affect chronic disease risk [41]. Exposure to microbiota in infancy at mismatched time points (too early or too late) may have long-term phenotypic effects. For example, kwashiorkor and severe malnutrition are associated with microbiota that is ‘underdeveloped’ for age in Malawi children [90]. Here, we extend the motivation for understanding the essential metabolic and immunological functions of commensal microbiota vital for maintaining health to the implications for behavioral phenotype, toward a more integrative developmental programming approach.

Milk bioactives and live bacteria are now increasingly integrated into clinical care, especially for infants in the form of nutritional supplements and medical treatment. Many neonatal intensive care units rely on donor milk sometimes augmented with human milk fortifier and probiotics, including lactobacilli and bifidobacteria, to reduce incidence of necrotizing enterocolitis (NEC) [91–94]. Although donor milk is pasteurized, killing the microbes, HMO remain intact and bioactive [95]. Currently, commercially available breast milk alternatives contain plant-derived oligosaccharides but not HMO [96]. Given the variation among mothers in HMO profiles and unexplored biobehavioral effects, selection of which HMO to incorporate into infant formulas is challenging. However, recently one HMO—disialyllacto-N-tetraose—was shown to reduce NEC in a rodent model [97].

Microbial treatments are also gaining traction for the remediation of behavioral and psychiatric symptoms. Recently, ‘psychobiotics’—live organisms, including bifidobacteria, that diminish symptoms of psychiatric illness—have garnered clinical attention [98]. In a rat model of depression, administration of bifidobacteria reversed depression-like behaviors, restored normal immune response and returned norephinephrine levels in the brainstem to baseline [46]. *Lactobacillus rhamnosus* colonization in mice reduced anxiety and affected expression of GABA receptors [98]. In humans, administration of *Lactobacillus helveticus* and *B.longum *reduces psychological distress and alleviates symptoms of depression compared with placebos [33]. More recently, prebiotic intake in human subjects has demonstrated decreased cortisol levels and decreased vigilance toward negative information [99]. As reviewed by Rook *et al.* [8] in this journal, the evaluation of the ‘Old Friends Hypothesis’ across diverse populations suggests that diverse microbial exposure during development modulates inflammation response over the lifetime. Downregulation of the inflammation response contributes to stress resilience, while exposure to less diverse microbes may increase risk for psychiatric disorder in adulthood through exaggerated inflammation response to social stress [8, 100].

While the evidence for bacterial therapeutics continues to accumulate, much remains unknown, especially regarding interventions during development when such manipulations exert greater phenotypic effects [30]. Aspects of this system may reflect push–pull dynamics between mother and offspring, a consideration rarely present in clinical discussions of neonatal health management. Applied microbiology in a clinical setting may precipitate unintended side effects for infant behavior, but also has the potential for targeted amelioration of undesired consequences from current medical practices.

## SUMMARY

The bioactive components in milk, produced by the mother, may be influencing the infant microbiota to shift the infant phenotype toward the mother’s optima for investment. Research in rodents, rhesus macaques and humans has already demonstrated biobehavioral effects of milk bioactives [36]. We hypothesize that other bioactives in milk, such as HMO, are also influencing behavioral phenotype and mediating maternal-offspring conflict and coordination through gut microbiota. As the first microbiota to colonize the infant originate from the mother [1, 4, 20] and are fed by mother’s milk [22], it appears that gut microbiota composition may be susceptible to maternal manipulation. This M2B2 system is extremely complex, encompassing a multitude of bacteria with more genes than the human genome [2], hundreds of HMO [16] and physiological and neurobiological systems of exquisite complexity. We predict that simple, singular answers to the phenotypic effects of mother’s milk and microbiota interactions are unlikely. An evolutionary perspective allows us to appreciate the essential tensions within the mother–infant dyad and recognize that the infant’s microbial ecology is a potential landscape for negotiating conflict and maintaining coordination. Among the many, many bacteria in the infant gut, may be lurking mother’s littlest helpers.
